# Anticoagulant effects, substance basis, and quality assessment approach of *Aspongopus chinensis* Dallas

**DOI:** 10.1371/journal.pone.0320165

**Published:** 2025-05-14

**Authors:** Jinzhou Fu, Guoli Zhang, Hongbing Peng, Zijie Xu, Yimei Liu, Keli Chen, Juan Li

**Affiliations:** 1 Hubei Key Laboratory of Resources and Chemistry of Chinese Medicine, School of Pharmacy, Hubei University of Chinese Medicine, Wuhan, China; 2 Hubei Shizhen Laboratory, Wuhan, China; 3 Medical Equipment Department, Huanggang Hospital of Traditional Chinese Medicine, Huanggang, China; Foshan University, CHINA

## Abstract

**Background:**

*Aspongopus chinensis* Dallas holds both medicinal and culinary significance in China. Notably, in regions such as Guizhou and Yunnan, it has been traditionally used as an ethnic remedy for treating various conditions, including stomach coldness, pain, kidney deficiencies, and impotence, among other ailments. This study aims to explore the chemical constituents and anticoagulant activity of *A. chinensis*.

**Methods:**

Ultraperformance liquid chromatography-tandem quadrupole time-of-flight mass spectrometry (UPLC-Q-TOF-MS) was employed for the UPLC fingerprint analysis. The isolated compounds underwent *in vitro* assays to their evaluate anticoagulant properties and effects on protein fibril activity. Network pharmacology was utilized to predict potential anticoagulant targets. Furthermore, animal experiments were conducted to measur coagulation factors and assess the *in vivo* anticoagulant activity.

**Results:**

Our findings indicate that 10 compounds were identified through UPLC-Q-TOF-MS analysis, with four compounds (uracil; 6-hydroxyquinolinic acid; 1,4-dihydro-4-oxoquinoline-2-carboxylic acid; delicatuline B) were isolated and identified from the n-butanol extract of *A. chinensis*. Furthermore, principal component analysis (PCA) and orthogonal partial least squares discriminant analysis (PLS-DA) of the UPLC fingerprint data revealed significant differences between *A. chinensis* and its similar insects. Notably, 1,4-dihydro-4-oxoquinoline-2-carboxylic acid has been identified as a potential standard reference substance for content determination. The isolated compounds showed anticoagulant properties, and the mRNA expression levels of *MMP9* and *PTGS2* were significantly reduced in LPS-induced RAW264.7 cells, further supporting the network pharmacology analysis. Animal experiments confirmed the potent anticoagulant effects of *A. chinensis*, likely associated with the intrinsic coagulation pathway and the inhibition of platelet aggregation,

**Conclusion:**

This research provides a quality assessment method for *A. chinensis*, and has also demonstrated its anticoagulant functions and substance basis

## Introduction

Insects, the most abundant group of animals in nature, inhabit a wide range of environments, including soil, plants, to air, water sources, corpses, and feces, and they even parasitize living animals and plants [1]. This abundance highlights their significant potential as medicinal agents and sources of nutritious food. Insects are functional organisms that contain bioactive compounds, characterized by high protein content, substantial amounts of lipids, vitamins, minerals, and fibers, and also notably contain chitin [2]. *Aspongopus chinensis* Dallas(*A. chinensis*), a species of pentatomid bug belonging to the Hemiptera family [3], is traditionally utilized as an ethnic remedy for the treatment of conditions such as stomach coldness, pain, kidney deficiencies, and impotence, among other ailments. Notably, in regions like Guizhou and Yunnan, *A. chinensis* is also valued as a culinary delicacy, holding both medicinal and culinary significance in folk medicine. Modern pharmacological research has shown that *A. chinensis* extracts have significant antibacterial, anticancer, analgesic, antifibrotic, and anticoagulant effects [4]. Noteworthy is the documented anticoagulant effect of *A. chinensis* decoctions, albeit the specific compound responsible for this effect remains unidentified. Ongoing research endeavors on *A. chinensis* have unveiled a spectrum of chemical components. For instance, Yan Yongming’s investigation yielded 59 compounds from *A. chinensis*, including 23 novel elements like alkaloid-amino acid hybrids, dopamine, and oligosaccharides, in addition to isolating 6 new and 3 known compounds [5]. Among these findings, a novel N-acetyl dopamine trimer was discovered by Yan Yongming et al., showcasing potent protective effects on kidneys [6]. Furthermore, certain sesquiterpenoids, lactams, and norepinephrine derivatives found in *A. chinensis* demonstrate protective properties against high glucose-induced Glomerular mesangial cells by safeguarding and inhibiting COX-2 expression [7]. Despite these advancements, the quality control of *A. chinensis* remains underexplored, marked by the absence of standardized reference substances and shortcomings like prolonged analysis times and limited sensitivity.

This experiment employed ultra-performance liquid chromatography-quadrupole time-of-flight mass spectrometry (UPLC-Q-TOF-MS) analysis, utilizing retention time, accurate mass, and MS/MS data [8], to explore the chemical composition of *A. chinensis*. Subsequently, the crude extract of *A. chinensis* underwent extraction and separation processes, and the structures of the compounds were elucidated using spectroscopic techniques such as nuclear magnetic resonance spectroscopy (NMR). Additionally, a Ultra Performance Liquid Chromatography (UPLC) fingerprint analysis method was developed for the quality assessment of *A. chinensis* and similar insects, including *Cyclopelta parva* Distant (*C. parva*) and *Megymenum inerme* H.-S (*H. inerme*). This method integrates techniques such as similarity analysis, hierarchical cluster analysis, principal component analysis (PCA), and orthogonal partial least squares discriminant analysis (PLS-DA). Furthermore, UPLC fingerprint analysis and content determination were conducted to support the quality control of *A. chinensis*. In parallel, the isolated components were assessed for their anticoagulant properties *in vitro*, and the extracts were tested *in vivo*. By integrating network pharmacology—a bioinformatics discipline adept at predicting the active ingredients and mechanisms of action of traditional Chinese medicine—this study investigated the anticoagulant mechanisms of *A. chinensis*’ chemical components [9]. Thus, this research contributes to the understanding of the anticoagulant effects, substance basis, and quality assessment approach of *A. chinensis*.

## Materials and methods

### Medicinal materials, reagents, and standards

Various batches of products were collected, which included *A. chinensis*, *Tessaratoma papillosa* Drury, *Erthesina fullo* Thunberg (*E. fullo*), *Megymenum inerme* H.-S (*M. inerme*1 ~ *M. inerme*4), and *Cyclopelta parva* Distant (*C. parva*1 ~ *C. parva*8). Professor Ke Li Chen from the Hubei University of Chinese Medicine identified each of the experimental samples through traditional taxonomical and morphological methods. Table 1 details the origins of the collected materials.

**Table 1:** Sample information of *Aspongopus chinensis* Dalla*s* and its similar insects.

No.	Name	Batch number	Distribution location
1	*Aspongopus chinensis* Dallas	ACD1–1003	Guizhou
2		ACD2–0506	Guizhou
3		ACD3–0502	Guizhou
4		ACD4–0509	Sichuan
5		ACD5–0319	Yunnan
6		ACD6–0559	Yunnan
7		ACD7–0508	Guangxi
8		ACD8–0507	Sichuan
9		ACD9–0510	Sichuan
10		ACD10–0512	Anhui
11		ACD11–0508	Guizhou
12		ACD12–0515	Guangxi
13		ACD13–0512	Yunnan
14		ACD14–0514	Guizhou
15		ACD15–1130	Sichuan
16		ACD16–0707	Vietnam
17	*Tessaratoma papillosa* Drury	TPD- 0508	Guangdong
18	*Erthesina fullo* Thunberg	EFT -0514	Guizhou
19	*Megymenum inerme* H.-S	MI1–0115	Yunnan
20		MI2–0502	Guangxi
21		MI3–0424	Anhui
22		MI4–0511	Yunnan
23	*Cyclopelta parva* Distant	CPD1–0520	Guizhou
24		CPD2–0501	Henan
25		CPD3–0812	Hunan
26		CPD4–0817	Sichuan
27		CPD5–1003	Henan
28		CPD6–1003	Henan
29		CPD7–0115	Sichuan
30		CPD8–0512	Jiangxi

Formic acid, either in chromatographic pure form or as methanol, was obtained from Shanghai Macklin. Petroleum ether and deuterated DMSO were also obtained from the same supplier. Methanol and acetone were procured from Hubei Shentshi Chemicals. Additionally, analytical-grade methanol and trifluoroacetic acid were sourced from China National Pharmaceutical Group. The compound 1,4-dihydro-4-oxoquinoline-2-carboxylic acid (compound **3**) was acquired from Shanghai Yuan Ye. Mitsubishi in Japan provided the MCI gel CHP 20P resin. while GE Healthcare supplied the Sephadex LH-20 gel column chromatography packing material. Furthermore, Merck KGaA supplied the reversed-phase silica gel RP-18 packing material, and Qingdao Ocean provided the thin-layer chromatography silica gel. Ultra-pure water was generated using a Water purifier ultra-pure water system.

Specific pharmaceutical products were procured, including Danshen Dropping Pills (DDP) from Tasly Pharmaceutical Group Co., Ltd. batch number 180302, and aspirin enteric-coated tablets from Bayer Healthcare Co., Ltd. batch number 85551191, were purchased. In terms of testing materials, the activated partial thromboplastin time (APTT) test kit (lot number F008-2), prothrombin time (PT) test kit (lot number F007), and thrombin time (TT) test kit (lot number F009) were sourced from Nanjing Jiancheng Biotech Institute of Engineering. Additionally, normal value coagulation quality control products were purchased from Chengdu Xiehe Biotechnology Co., Ltd.

DMEM High Sugar Medium (Solarbio, 20210325); Fetal Bovine Serum (FBS, Zhejiang TianHang Biotechnology Co. Ltd., 20080502); CCK8 (Biosharp, P23000015343); Lipopolysaccharides (LPS) (Sigma, 0000185317); Trizol (Tiangen, X1215); Reverse Transcription Kit (Abtek, 962329F26W06); Real Universal Fluorescence Quantification Reagent SYBR Green (Abtek, 962303F08W09); The primers were synthesized by Shanghai Bioengineering Co.

### Cells and animals

The mouse monocyte macrophage cell line RAW264.7 was generously provided by the Department of Immunology, Tongji Medical College, Huazhong University of Science and Technology.

The experimenters passed the Hubei Provincial Laboratory Animal Practitioner Professional and Technical Examination and possess the capability to handle experimental animals. Animal procedures were approved by the Animal Ethics Committee of Hubei University of Traditional Chinese Medicine (license number: **SYXK (E) 2017–0067**). All animals were housed under standard laboratory conditions (temperature, 23–25°C; humidity, 50 ± 5%) with free access to water and were acclimatized with feeding for one week. The study included 48 male Sprague-Dawley (SD) rats, weighing 220–300 g; 60 male Kunming (KM) mice, weighing 18–20 g; and New Zealand rabbits, all provided by the Animal Experiment Center of Hubei University of Traditional Chinese Medicine.

Mice were anesthetized with 1% sodium pentobarbital (50 mg·kg^-1^) via intraperitoneal injection and euthanized with an overdose post-experiment. Rats were anesthetized with 1% sodium pentobarbital ((40 mg·kg^-1^) intraperitoneally and euthanized after blood collection from the abdominal aorta. Rabbits were anesthetized with 0.7% sodium pentobarbital via intravenous injection in the ear margin and euthanized at the end of the experiment.The animal study was reviewed and approved by the Center Laboratory of Chinese Medicine, Hubei University of Chinese Medicine (**HUCMS202211003**).

### Sample preparation

The dried whole insects were ground into a powder at 60°C, and precisely weighed insect powder (1.0000 g) was added to 25 mL of methanol, sealed, and sonicated for 20 min. Following this, the mixture was allowed to stand and filtered, with the process being repeated once, and the two filtrates were combined. The resulting mixture was extracted twice with petroleum ether (60–90°C), using 100 mL each time. Subsequently, the alcohol phase was dried in a vacuum drying oven, dissolved in 10 mL of methanol, filtered through a 0.22 μm microporous membrane, and set aside for later use. Concurrently, compound **3** was employed as the reference substance, precisely weighed, dissolved in methanol, and prepared into solutions with concentrations of 0.063, 0.095, 0.104, 0.155, and 0.207 mg·mL^-1^.

Compound **3** was isolated and identified from the n-butanol extract of wormwood through a series of chromatographic separations, including microporous resin column chromatography, LH-20 gel column chromatography, C18 reverse-phase silica gel column chromatography, and preparative liquid chromatography. Additionally, compound **1** (uracil), compound **2** (6-hydroxykynurenic acid), and compound **4** (delicatuline B) were prepared. Heparin sodium (10 U·mL^-1^) was dissolved in 10% dimethyl sulfoxide (DMSO) to create a 1 mg·mL-1 test solution.

Urokinase standard (50,000 U·mL^-1^) was used to prepare solutions with concentrations of 100, 80, 60, 40, and 20 U·mL^-1^ using a 0.01 mol·L^-1^ phosphate buffer at pH 7.4, resulting in a series of five distinct concentration standard solutions [10].

### UPLC-Q-TOF-MS and UPLC analysis

The UPLC-Q-TOF-MS analyses were performed using a Waters Acquity I-Class UPLCTM liquid chromatograph, which featured a binary solvent delivery system and a photodiode array detector. A Guangzhou Lichen Biological Technology Co., Ltd. Welch Ultimate TM UPLC XB-C18 column (2.1mm × 100 mm, 1.8μm) was utilized in this study, with the column temperature maintained at 40°C. Elution was carried out using a gradient mobile phase consisting of 0.1% formic acid solution (A) and methanol (B). The UPLC elution conditions were optimized as follows: 0–2 min, 10% B; 2–3.5 min, 10%-20% B; 3.5–5 min, 20% B; 5–6.5 min, 20%-27% B; 6.5–7.5 min, 27% B; 7.5–17.5 min, 27%-94% B; 17.5–18.5 min, 94% B; 18.5–20 min, 94%-100% B; 20–24 min, 100% B; 24–25 min, 100%-10% B. For the mass spectrometric analysis, a Waters Xevo-G2XSQTOF high-resolution mass spectrometer equipped with an electrospray ionization source (ESI) was utilized in both positive and negative ion modes. The electrospray source and the desolvation gas were operated at temperatures of 100°C and 500°C, respectively, with a desolvation gas flow rate of 500 L·h^-1^. The ESI capillary voltage was set at 2500 V, and the cone voltage was set at 40 V. The scan range was set from 50 to 1200, with argon serving as the collision gas during the analysis. In low-energy scan mode, a voltage of 6 eV was employed, whereas in high-energy scan mode, the voltage ranged from 30 to 40 eV. The analysis was facilitated using Masslynx V4.1 as the operating system with a flow rate maintained at 0.3 mL·min^-1^ and an injection volume of 2 μL. The detection wavelength was set at 254 nm to ensure accurate and reliable results.

The UPLC analysis was conducted using an Agilent 1260 Infinity II high-performance liquid chromatograph equipped with an Agilent ZORBAX Eclipse Plus C18 column (2.1 × 50 mm, 1.8-Micron) at a column temperature of 40°C. A gradient mobile phase composed of 0.05% trifluoroacetic acid solution (A) and methanol (B) was employed for elution. The optimized elution conditions for the UPLC were structured as follows: from 0 to 2 min, 10% B; 2 to 3.5 min, 10% to 20% B; 3.5 to 5 min, 20% B; 5 to 6.5 min, 20% to 27% B; 6.5 to 7.5 min, 27% B; 7.5 to 12 min, 27% to 50% B; 12 to 20 min, 90% B; 20 to 23 min, 90% to 100% B; 23 to 30 min, 100% B; and from 30 to 31 min, 100% to 10% B. The analysis was monitored at a detection wavelength of 254 nm, with a precisely controlled flow rate of 0.4 mL·min^-1^, and an injection volume of 1 μL.

### Isolation and extraction of components

**Fig 1:**
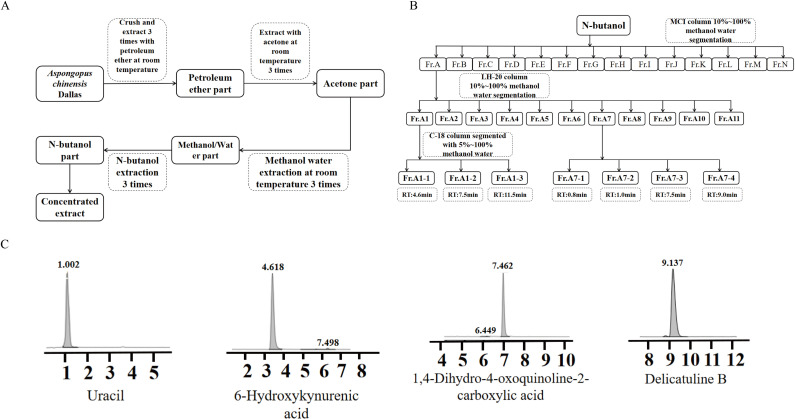
Extraction and separation flowchart of *Aspongopus chinensis* Dallas(A, B) and the liquid-phase diagram of small molecule compounds from *A. chinensis* (C). Note: Uracil(compound **1**); 6-hydroxykynurenic acid(compound **2**); 1,4-dihydro-4-oxoquinoline-2-carboxylic acid(compound **3**); delicatuline B(compound **4**).

There are reports in the literature indicating that the total activity of extracts extracted with sodium chloride, petroleum ether, acetone, and water is higher [11]. Therefore, we employed a multi-solvent extraction method to obtain the most active components from *A. chinensis*. The insect was crushed into coarse powder and sequentially extracted at room temperature with petroleum ether, acetone, and 50% methanol-water. Each solvent extraction was performed three times with 25 L for 48 h. The combined 50% methanol-water extracts were concentrated to yield a crude extract weighing 350 g, which was then subjected to extraction with n-butanol three times, yielding a n-butanol fraction of 35 g. The extraction and separation process is illustrated in Fig 1A and 1B. The n-butanol fraction was further purification through column chromatography on MCI gel CHP 20P, utilizing a gradient elution of methanol/water (10:90, 20:80, 30:70, 40:60, 50:50, 60:40, 70:30, 80:20, 90:10, 100:0). TLC/HPLC was used for detection, and fractions with similar profiles were combined, yielding 14 sub-fractions labeled Fr.A-N. Specifically, fraction Fr.A (3 g) was then subjected to Sephadex LH-20 column chromatography using a gradient elution of methanol/water, resulting in 11 sub-fractions labeled Fr.A1-11. The detailed process is presented in Fig 1A and 1B for reference.

Fraction Fr. A1 (250 mg) was further subjected to reverse-phase C18 column chromatography using a gradient elution of methanol/water to obtain compounds **2** (4.5 mg, tR = 4.6 min), **3** (11.9 mg, tR = 7.5 min). Fraction Fr. A7 (1,173.8 mg) was subjected to reverse-phase C18 column chromatography using a gradient elution of methanol/water to obtain **1** (30 mg, tR = 1.0 min), **3** (30 mg, tR = 7.5 min), and **4** (2 mg, tR = 9.1 min). Fraction Fr.F (4 g) was processed using LH-20 gel column chromatography using a gradient elution of chloroform/methanol (50:50, 40:80, 30:70, 20:80, 10:90, 0:100). HPLC was employed for detection, and fractions with similar profiles were combined.

### Anticoagulation experiment

PT and APTT were assessed utilizing reagent kits obtained from the Nanjing Jiancheng Biotech Institute of Engineering, in accordance with the manufacturer’s instructions. Coagulation time was recorded by a semi-automatic coagulation analyzer, which measured the duration from sample addition until the cessation of bead oscillation. Additionally, the coagulation times of ultrapure water and buffer solution were also determined during the experiment.

### Fibrinolytic activity assay using agarose-fibrinogen plate method

To prepare the agarose-fibrinogen plate, 1 g of agarose was placed in a 150 mL conical flask, and 90 mL of 0.01 mol·L^-1^ phosphate buffer solution (pH 7.4) was added. The mixture was heated to boiling to completely dissolve the agarose and then cooled to 50–60 °C. Following this, 6 mL of preheated bovine blood fibrinogen (Shanghai Yuanye, containing 90 mg) was quickly added and mixed in. Following this step, 30 mL of the mixture was transferred to a 9 cm diameter culture dish. Subsequently, 0.5ml of the pre-prepared thrombin solution (containing 1 mg) and 0.5ml of the bovine blood fibrinogen plasminogen solution (containing 1 mg) were added, mixed rapidly, and allowed to stand for half an hour after solidification. Finally, the solidified mixture was perforated with a 4 mm punch, creating 7–8 holes per plate and 3 plates prepared for each experiment [12].

The plate was incubated at 37 °C in a humid environment for 18 h after aspirating 20 μL of both the urokinase standard and sample solutions using a pipette and depositing them onto the same plate. After incubation, the diameter of the lysis ring was measured with a vernier caliper, and the lysis area was then calculated. Subsequently, the logarithm of the lysis area was then plotted against the logarithm of the urokinase standard enzyme activity, facilitating the calculation of the potency of the sample [13].

### Network pharmacology prediction

The Swiss Target Prediction database (http://www.swisstargetprediction.ch/) was queried for the targets of 4 compounds: compound **1**, compound **2**, compound **3**, and compound **4**. The predicted target protein names were then converted to corresponding gene names using the Uniprot database (http://www.uniprot.org/).

The GeneCards database (https://www.genecards.org/) was searched for anticoagulation-related targets. The identified anticoagulation-related protein targets were found to intersect with protein targets related to *A. chinensis*. The obtained protein-protein interaction Protein-Protein Interaction (PPI) network was generated using the online STRING 12.0 software (https://string-db.org/cgi/input.pl) [14]. The potential core target proteins were subjected to Gene Ontology (GO) function and KEGG pathway [15] enrichment analysis using the DAVID 6.8 database (https://david.ncifcrf.gov/). A P-value of less than 0.05 was considered statistically significant. Based on 4 components and 32 targets, a “component-target-pathway” network diagram was constructed using Cytoscape 3.7.2 software.

### CCK-8 assay

RAW264.7 cells were cultured in a 37°C incubator with 5% CO₂, using high-glucose DMEM medium supplemented with 10% fetal bovine serum and 1% double antibody. Cells in the logarithmic growth phase were harvested and inoculated into 96-well plates at a density of 1 × 10⁴ cells per well. After 24 h, the medium was replaced with DMEM containing various concentrations of compounds, and *A. chinensis* extract. The cells were then incubated for an additional 24 h. After incubation, the supernatant was discarded, and 10 μL of CCK-8 solution and 90 μL of fresh medium were added to each well. The plates were incubated for 30 min, and the absorbance (A) at 570 nm was measured using a microplate reader (BioTek Instruments, Inc, United States).

### Reverse transcription-polymerase chain reaction (RT-PCR)

After 24 h treatment, cells were collected, and total cellular RNA was extracted using TRIzol reagent. cDNA was synthesized according to the instructions provided in the reverse transcription kit, and qRT-PCR analysis was performed. A 20 μL reaction system was used, with the PCR amplification conditions set as follows: 95 °C for 15 min, followed by 40 cycles of 95 °C for 10 s, 60 °C for 40 s, and an extension at 72 °C for 32 s. The relative expression of mRNA was calculated using the 2^-ΔΔCT^ method. The primer sequences were as follows: *β-actin*: forward CCTGACTGACTACCTCATGAAG, reverse GACGTAGCACAGCTTCTCCTT; *MMP9*: forward AGTATCTGTATGGTCGTGGGCTCTAAG, reverse GGAGGTGCTGTCGGGCTGTG; *PTGS2*: forward ATTCCAAACCAGCAGACTCATA, reverse CTTGAGTTTGAAGTGGTAACCG.

### Determination of coagulation time in KM mice by capillary method

60 KM mice were randomly divided into 6 groups of 10 mice each, comprising a blank control group (normal saline), an aspirin control group (0.026 g·kg^-1^), and a DDP control group (0.1053 g·kg^-1^), along with high, medium, and low dose administration groups of *A. chinensis* extract (0.182, 0.091, and 0.0455 g·kg^-1^). Administration was conducted through gavage at a dosage of 0.01 mL·g^-1^ continuously for 7 d. Subsequently, 1 h after the final administration, blood collection was initiated by inserting a glass capillary tube into the posterior canthus venous plexus of the mouse eye. The collected blood filled the glass capillary vessel, which was then placed on a table for timing. By breaking one end approximately 0.5 cm every 30 s, and observing the appearance of coagulation filaments at the broken point, the CT was recorded. The mice were positioned in a holder with their tails vertically aligned, and the tail was then cut 3 mm from the tip. Blood was gently extracted from the tail tip using filter paper every 30 s until no further bleeding occurred. The time interval from tail cutting to the cessation of bleeding was documented as the BT.

### Determination of coagulation function in SD rats

60 SD rats were randomly assigned to 6 groups consisting of 10 rats each. The groups included a blank control group (administered normal saline), an aspirin control group (0.018 g·kg^-1^), and a DDP control group (0.0729 g·kg^-1^). Additionally, there were 3 administration groups for *A. chinensis* extract, which received high, medium, and low doses (1.26, 0.63, 0.315 g·kg^-1^). Administration was carried out via gavage at a dosage of 0.01mL·g^-1^ continuously for 7 d. Following the last administration, the rats were anesthetized through intraperitoneal injection of sodium pentobarbital after 1 h, and blood samples were collected from the abdominal aorta using an EP tube containing a 3.2% sodium citrate anticoagulant solution. The collected samples were then centrifuged to obtain the upper plasma. Subsequently, a hemagglutometer was employed to measure APTT, TT, and PT.

### Rabbit platelet aggregation test

To collect blood from the ear margin veins of male New Zealand rabbits, an EP tube containing a 3.2% sodium citrate anticoagulant solution must be used. The collected blood should be gently mixed by inverting the tube. Subsequently, centrifuge the blood at 800 rpm for 10 min to obtain the upper layer of platelet-rich plasma (PRP). The remaining plasma in the lower layer should then be centrifuged at 3000 rpm for 15 min to collect the upper layer of platelet-poor plasma (PPP). For the experimental setup, the control group comprises 280 μL PPP and 10 μL distilled water. The administration group consisted of 280 μL PRP mixed with different concentrations of *A. chinensis* extract (0.3125, 0.625, 1.25, 2.5 g·mL^-1^). Additionally, the positive control group involves mixing 280 μL PRP with a 10 mg·mL^-1^ aspirin solution.

### Statistical analysis

The original data obtained from HPLC analysis were peak aligned, selected, and normalized through the “TCM Chromatographic Fingerprint Similarity Evaluation System (2012 Edition)” to generate a matrix (Rnull) containing peak area and retention time (t). Hierarchical cluster analysis and principal component analysis were performed using SPSS 21.0 software. Subsequently, PLS-DA analysis was conducted using the SIMCA 16 software, with the common peak areas of the samples from *A. chinensis*, *C. parva*, and *M. inerme* as the variables. The VIP value was utilized to identify the key ingredients that significantly influenced the variations in medicinal ingredients among the samples. Ingredients with VIP values greater than 1 were regarded as having substantial contributions [16].

## Results

### UPLC-Q-TOF-MS and UPLC analysis

The evaluation of precision, stability, repeatability, and common peak area of the samples was conducted according to the established operating method by calculating the retention time (t). The results indicated that the relative standard deviation (RSD) of the common peak retention time ranged from 0.04% to 0.45%, 0.04% to 0.48%, and 0.05% to 0.67%, respectively. Additionally, the relative peak area RSD ranged from 0.82% to 3.4%, 1.4% to 2.9%, and 0.35% to 2.6%, respectively. These findings suggested that the instrument exhibited good precision, the stability of the test sample solution remained optimal within a 24 h timeframe, and the method demonstrated excellent repeatability.

**Fig 2:**
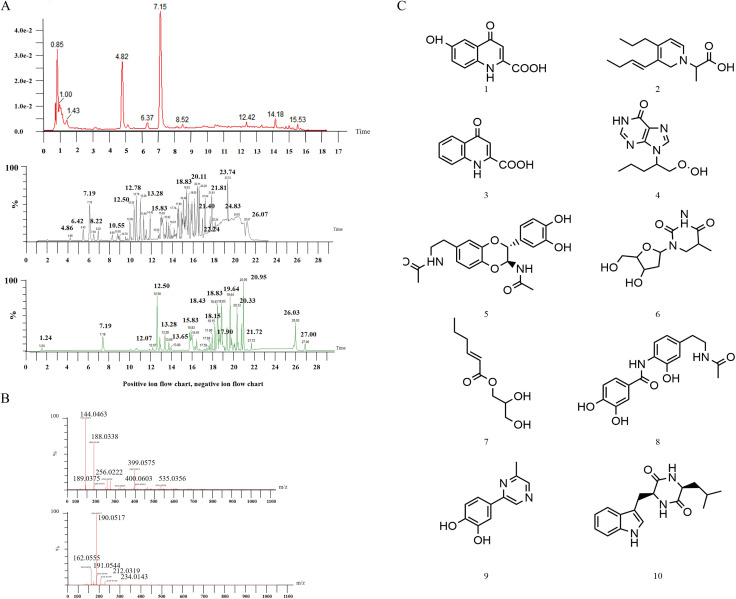
The UPLC-Q-TOF-MS chromatogram of *Aspongopus chinensis* Dallas in positive and negative ion modes (A). Mass spectrum in positive and negative ion modes at retention time of 7.19min. (B). Structural formula of the identified compounds from *A. chinensis* by UPLC-Q-TOF-MS (1-10) (C).

The total ion chromatogram data for *A. chinensis* in both positive and negative ion modes were collected and analyzed, resulting in the identification of 10 components (Fig 2A). At a retention time of 7.19 min, the characteristic peak of the compound was observed in both positive ion, 190.0517 [M + H]^+^, and negative ion, 188.0338 [M-H]^-^ modes (Fig 2B), indicating a molecular weight of 189. The compound was inferred to be compound **3** based on the characteristic fragment ions m/z 162.0555 [M + H-CO]^+^ and m/z 144.0463 [M-H-COO]^-^ according to the literature review results [17]. Through comparison with the standard, the compound was further confirmed to be compound **3**. Additional compounds corresponding to the chromatographic peaks are listed in Table 2 and Fig 2C.

**Table 2:** The identified compounds from *Aspongopus chinensis* Dallas by UPLC-Q-TOF-MS.

Number	Retention time (min)	M^-^	M^+^	Molecular weight	Name	Molecular formula
1	4.865	204.0280 [M-H]^-^	206.0443 [M + H]^+^	205	6-Hydroxykynurenic acid	C_10_H_7_NO_4_
2	6.42		250 [M + H]^+^	249	——	C_15_H_23_NO_2_
3	7.19	188.0338	190.0517 [M + H]^+^	189	1,4-Dihydro-4-oxoquinoline-2-carboxylic acid	C_10_H_7_NO_3_
4	8.59		251.1151 [M + H]^+^	250	Asponguanine A	C_11_H_14_N_4_O_3_
5	10.64	384.9334 [M-H]^-^	409.1372 [M + Na]^+^	386	Trans-2-(3’, 4’-dihydroxyphenyl) -3-acetylamino-6-(N-acetyl-2″-aminoethyl) -1, 4- Benzodioxaneor trans-2-(3’, 4’-dihydroxyphenyl) -3-acetylamino-7-(N-acetyl-2″-aminoethyl) -1, 4- benzodioxane	C_20_H_22_N_2_O_6_
6	12.23	243.1227 [M-H]^-^	267.1208 [M + Na]^+^	244	5,6-Dihydrothymidine	C_10_H_16_N_2_O_5_
7	12.50	187.0971 [M-H]^-^	211.0974 [M + Na]^+^	188	2-Hexenoic acid-2,3-dihydroxypropyl ester	C_9_H_16_O_4_
8	14.21		225.1109 [M + Na]+	202	Aspongpyrazine A	C_11_H_10_N_2_O_2_
9	15.67	329.2336 [M-H]^-^	353.2301 [M + Na]^+^	330	Aspongamides C	C_17_H_18_N_2_O_5_
10	18.83	298.2470 [M-H]^-^	322.2454 [M + Na]^+^	299	Cyclo (L-leucyl-L-tryptophyl)	C_17_H_21_N_3_O_2_

### Isolating and extracting small molecules from *A. chinensis*

**Fig 3:**
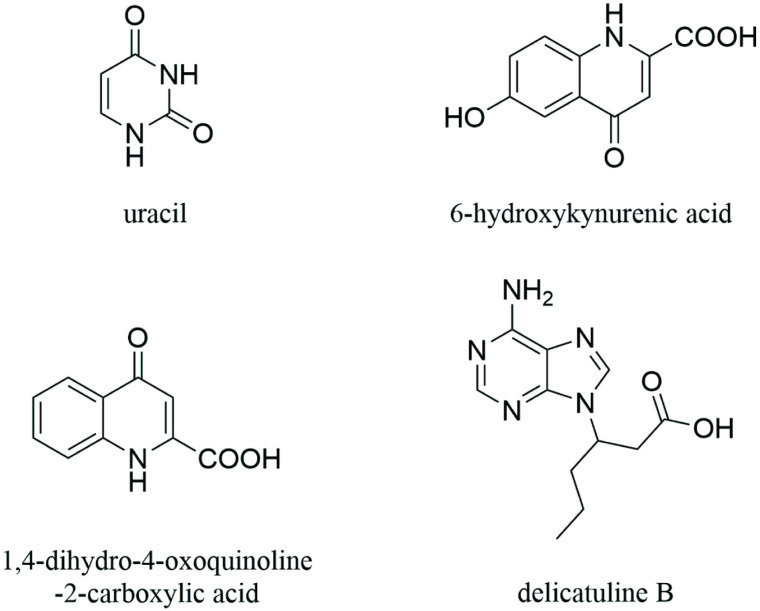
Structural formula of isolated compounds from *Aspongopus chinensis* Dallas. Note: Uracil(compound **1**),6-hydroxykynurenic acid(compound **2**),1,4-dihydro-4-oxoquinoline-2-carboxylic acid(compound **3**),and delicatuline B(compound **4**).

Four compounds were isolated from *A. chinensis* through a series of purification methods, including the micro-porous resin column, LH-20 gel column, C18 reversed-phase silica gel column, and preparative liquid chromatography. An Agilent DD2400-MR nuclear magnetic resonance spectrometer in conjunction with an Agilent QQQ mass spectrometer was employed for structural analysis. Based on mass spectrometry data, compounds **1**, **2**, **3**, and **4** were identified as uracil, 6-hydroxykynurenic acid, transtorine, and delicatuline B, respectively, with molecular weights of 112, 205, 189, and 250, correspondingly. To confirm the structures of these compounds, spectral data were compared with existing literature were utilized [18–21]. The structural formulas of the identified compounds can be found in Fig 3. Detailed NMR information is available in S1 File, S2 Fig for further examination.

### Similarity evaluation and principal component analysis through UPLC fingerprint

**Fig 4:**
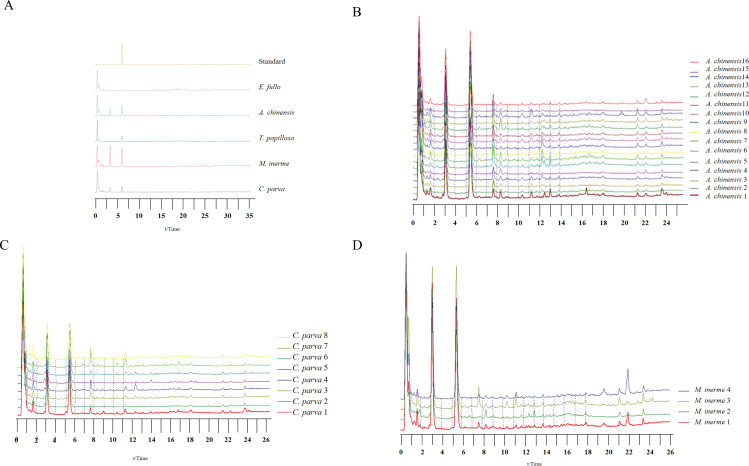
Control chromatograms of *Aspongopus chinensis* Dallas and its similar insects (A) and fingerprints of *A. chinensis* (1-16), *Cyclopelta parva* Distant (1-8), and *Megymenum inerme* H-S(1-4) (B, C, D).

A total of 30 batches of samples, including *A. chinensis*, *C. parva*, *M. inerme*, *T. papillosa*, and *E. fullo*, were analyzed using chromatographic analysis. The chromatograms for the various sample types, along with the standard samples are presented in Fig 3A. Notably, Fig 4A illustrates significant differences in the liquid phase chromatograms between *T. papillosa* and *E. fullo*. Conversely, the UPLC characteristic peaks observed in *A. chinensis*, *C. parva*, and *M. inerme* samples closely resemble each other, as demonstrated in Fig 4B, 4C, and 4D.

**Fig 5:**
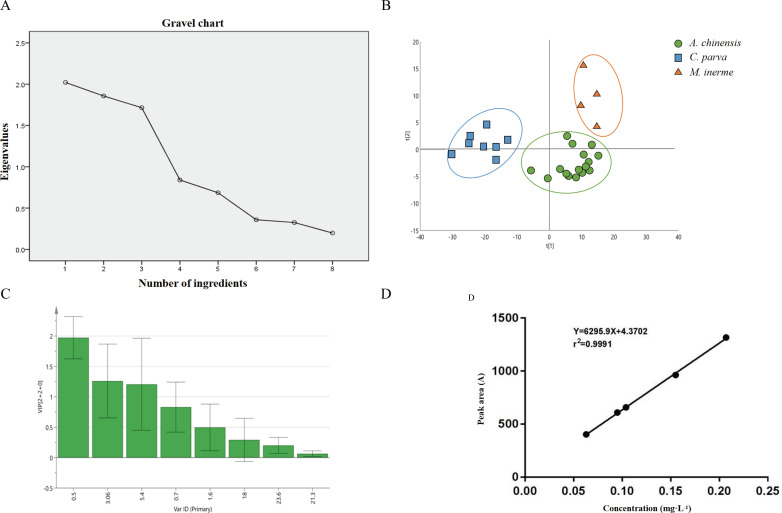
Principal component analysis scree plot (A), PLS-DA score plot (B), VIP value of 8 peaks of *Aspongopus chinensis* Dallas and its similar insects (C) and the standard curve of 1,4-dihydro-4-oxoquinoline-2-carboxylic acid (compound 3) (D).

Principal component analysis was conducted using SPSS 21.0 software to analyze 16 batches of *A. chinensis* samples, 8 batches of *C. parva* samples, and 4 batches of *M. inerme* samples for similarity based on the reference chromatogram [22]. The results indicate good similarity among the samples, with detailed findings presented in S3 Table. The analysis reveal that the first three principal components [23], which account for 69.916% of the information from 8 common components, can effectively summarize the original data. This is further illustrated in Table 3. Additionally, the scree plot illustrating the analysis results is depicted in Fig 5A.

**Table 3:** Principal component characteristic values and cumulative variance contribution rates.

Ingredient	Eigenvalues	Variance contribution rate (%)	Cumulative variance contribution rate (%)
1	2.021	25.263	25.263
2	1.857	23.215	48.478
3	1.715	21.438	69.916

The cumulative contribution rates of the first three principal components of *A. chinensis*, *C. parva*, and *M. inerme* samples reached 69.916%, indicating that the first three principal components can summarize most of the original data’s information [24].

The PLS-DA analysis results indicated a distinct difference between *A. chinensis* and *C. parva* as well as *M. inerme*, highlighting variations in their chemical compositions, as illustrated in Fig 5B. Three major contributing components were identified using the VIP value to select characteristic components influencing the differences in medicinal components, with a VIP > 1 as the screening standard. These components, specifically chromatographic peaks with retention times of 0.5 min, 3.06 min, and 5.4 min, were found to be consistent with the principal component analysis results. Through liquid chromatography-mass spectrometry, it was confirmed that the peak substance with a retention time of 3.06 min had a molecular weight of 205 (compound **2**), while the substance with a retention time of 5.4 min had a molecular weight of 189, identified as compound **3** (Fig 5C). Both the principal component analysis and PLS-DA analysis demonstrated a distinguishable difference among *A. chinensis* , *C. parva* and *M. inerme*, suggesting variations in their chemical compositions.

### Content determination

The standard curve and regression equation were plotted by using the concentration of the reference substance as the abscissa and the peak area as the ordinate. This analysis resulted in a regression equation of Y = 6295.9X-4.3702 and an r-squared value of 0.9991, indicating a strong linear relationship within the concentration range of 63 ~ 207 μg·mL^-1^. The data presenting these findings can be observed in Fig 5D. Subsequently, the percentage content of compound **3** in the chromatographic data of each batch of *A. chinensis* samples was determined by substituting the peak area into the previously established linear equation. Details of these calculations and outcomes can be found in S4 Table. The content of compound **3** in *A. chinensis* is significantly higher than that in *M. inerme* and *C. parva*, while the content in *M. inerme* is slightly higher than that in *C. parva*.

Previous studies on the chemical composition differences between *A. chinensis* and its similar products were unclear, and there was a lack of corresponding standards for content determination. This experiment, for the first time, used the compound compound **3** as the standard reference substance for both the fingerprint profile and content determination of *A. chinensis*, providing a standard for the quality control of this insect.

### Effect of anticoagulant and fibrinolytic activity *in vitro*

**Fig 6:**
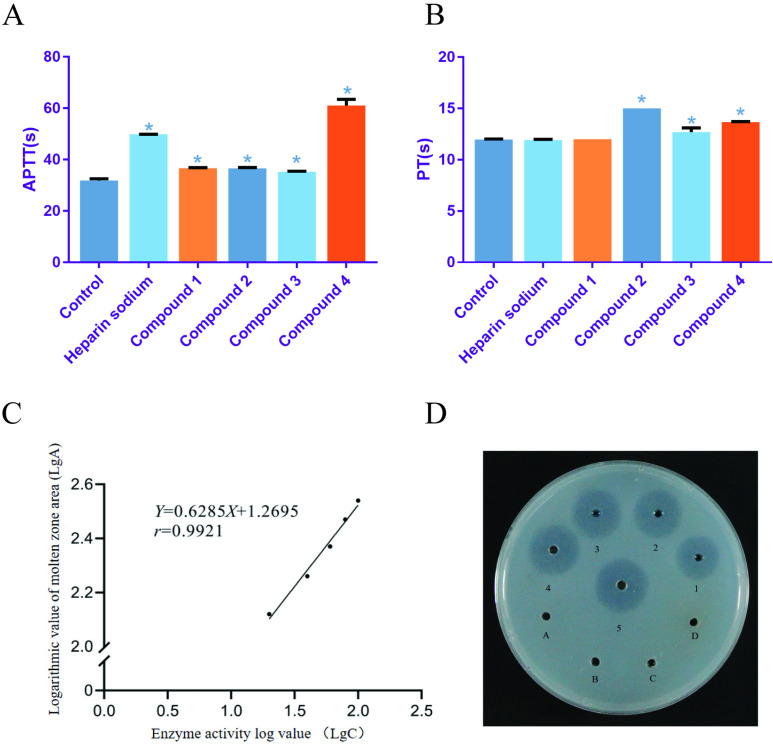
Anticoagulant activity of compounds from *Aspongopus chinensis* Dallas(A,B). **Standard curve of urokinase activity** (C), and fibrin plate dissolution circle (D). Note: Compounds **1** (uracil), **2** (6-hydroxykynurenic acid), **3** (1,4-dihydro-4-oxoquinoline-2-carboxylic acid), and **4** (delicatuline B); * indicates comparison with control group, * Correlation is significant at the 0.05 level; ** Correlation is significant at the 0.01 level. A, B, C, D circles (**D**) are compounds **1**, **2**, **3**, **4**; 1 ~ 5 represents urokinase activity of 20, 40, 60, 80, 100 U·mL^-1^ respectively.

Significant differences in APTT and PT values were observed during the *in vitro* anticoagulant experiments and fibrinolytic activity assays conducted on the four compounds extracted from *A. chinensis* (Fig 6A and 6B). Compounds **1**, **2**, **3**, and **4** exhibited higher APTT values compared to the blank group (10% DMSO). Compounds **2**, **3**, and **4** showed elevated PT values as well, indicating their significant anticoagulant properties in comparison to the blank group. Particularly, compound **4** displayed superior anticoagulant effects to the positive control, sodium heparin (10 U·mL^-1^). These findings suggest that compounds **1**, **2**, **3**, and **4** possess *in vitro* anticoagulant effects, underscoring the potential therapeutic significance of compound **4** as an effective anticoagulant agent.

The standard curve equation for the urine kinase was Y = 0.6285X + 1.2695 (Fig 6C), with a correlation coefficient (r) of 0.9921, demonstrating a strong linear relationship over the range of 20–100 U·mL^-1^. The urine kinase standard curve was constructed following the procedure outlined in reference [25]. Analysis of the solubilization ring diagram (Fig 6D) indicated that none of the four compounds exhibited any fibrinolytic activity.

### Network pharmacology analysis

**Fig 7:**
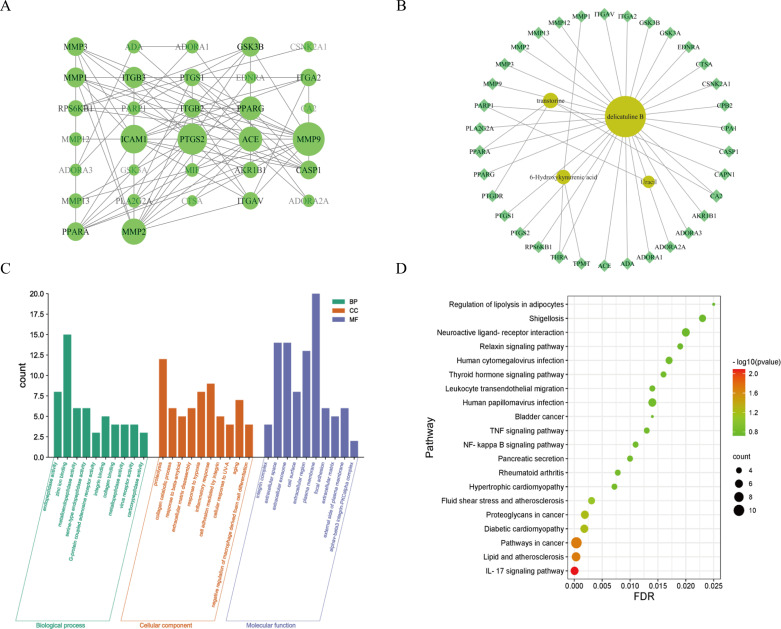
Protein Protein-Protein Interaction (PPI) network diagram (A); “component-target” network diagram (B); Gene Ontology (GO) functional enrichment analysis histogram (C); Kyoto Encyclopedia of Genes and Genomes (KEGG) pathway enrichment analysis bubble diagram (D).

Using “anticoagulant” and “thrombus” as keywords, 1659 disease targets were retrieved. Network pharmacology analysis was carried out by first selecting target proteins from the database, removing duplicates, and eventually identifying 105 target proteins associated with the four compounds. The overlap of disease targets and ingredient targets led to the identification of 38 potential target proteins. These potential targets were then used to construct a protein interaction network, which was visualized using Cytoscape 3.7.2, resulting in 32 action target proteins illustrated in Fig 7A. The selection of core target proteins was based on a degree greater than 10, which included *MMP9*, *PTGS2*, *ICAM1*, *MMP2*, *ACE*, and *PPARG*. Subsequently, GO function and KEGG pathway enrichment analyses were conducted on the 32 potential core target proteins. The results of both analyses were found to be statistically significant with *P* < 0.05 and *FDR* < 0.05. The GO function enrichment analysis identified a total of 66 GO entries, which can be viewed in S5 Table and depicted in Fig 7B. Likewise, KEGG enrichment analysis revealed six pathways, shown in Table 4 and illustrated in Fig 7C. Notably, among these pathways, lipid, and atherosclerosis were found to be linked to inflammation-related cardiovascular diseases, suggesting a potential association between the anticoagulant effect of *A. chinensis* and these pathways.

**Table 4:** Pathway enrichment analysis of main targets.

ID	Gene function	Number of genes	*P* value	FDR
hsa04657	IL-17 signaling pathway	6	0.000021	0.0030
hsa05200	Pathways in cancer	10	0.000083	0.0053
hsa05417	Lipid and atherosclerosis	7	0.000110	0.0053
hsa05415	Diabetic cardiomyopathy	6	0.000790	0.0240
hsa05205	Proteoglycans in cancer	6	0.000820	0.0240
hsa05418	Fluid shear stress and atherosclerosis	5	0.001600	0.0380

A “component-target” network diagram was constructed based on the four components and 32 core target proteins, as illustrated in Fig 7D. The connectivity of compound **4** with target proteins was found to be the highest, indicating that compound **4** primarily interacts with core target proteins such as *MMP9* to produce anticoagulant effects. This observation supports the notion that compound **4** is the most potent component, which is consistent with the results of the anticoagulant experiments. The degree values of pivotal target proteins *MMP9* and *PTGS2* in the PPI network highlight their importance as core targets.

### The effects of components on viability and *PTGS2* and *MMP9* mRNA expression in RAW264.7 cells

**Fig 8:**
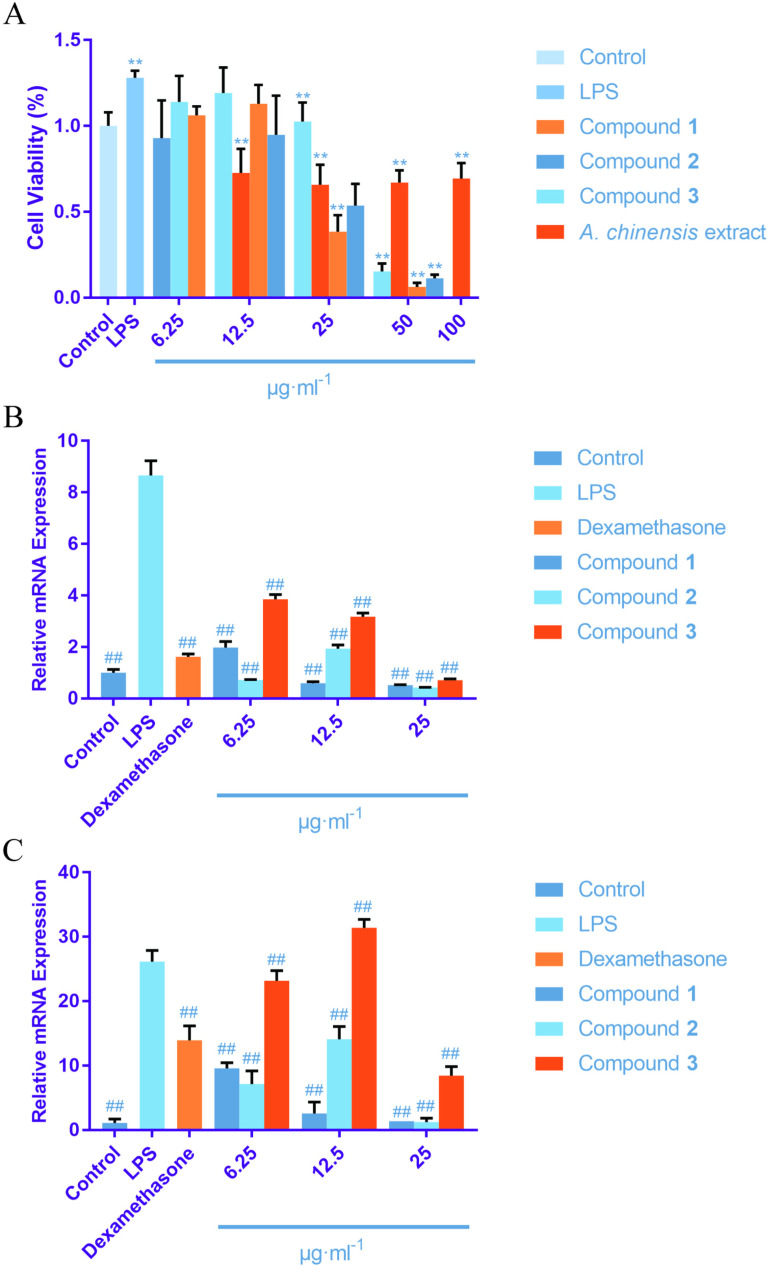
A illustrates the survival rates of CCK-8 experimental cells treated with compounds 1 (uracil), 2 (6-hydroxykynurenic acid), 3 (1,4-dihydro-4-oxoquinoline-2-Carboxylic acid), and extracts from *Aspongopus chinensis* Dallas. **B and C demonstrate the effects of compounds 1, 2,and 3 on the expression levels of the *PGST2* (B) and *MMP9* (C) genes.** Note: * indicates comparison with control group; * Correlation is significant at the 0.05 level; ** Correlation is significant at the 0.01 level; # indicates comparison with model group; # Correlation is significant at the 0.05 level; ## Correlation is significant at the 0.01 level.

Cells exposed to compound **1** and **3** at concentrations below 12.5 μg·mL^-1^ had a survival rate exceeding 90%, indicating no significant cytotoxicity. Similarly, compound **2** showed no apparent cytotoxic effects at concentrations below 50 μg·mL^-1^. Additionally, cells exposed to *A. chinensis* extract, which exhibited slight cytotoxicity at all tested concentrations, maintained a survival rate above 60%. See Fig 8A.

As shown in Fig 8B and 8C, the mRNA expression levels of both *PTGS2* and *MMP9* in the cells of the model group were significantly higher than those in the control group (*P* < 0.01). In contrast, in the positive control (dexamethasone) group, the mRNA expression levels of *PTGS2* and *MMP9* were significantly lower (*P* < 0.01). Compared to the model group, the mRNA expression levels of *MMP9* and *PTGS2* were significantly reduced in the groups treated with various concentrations of compound **1**, **2** and **3** (*P* < 0.01).

### Anticoagulant activity of *A. chinensis*

**Fig 9:**
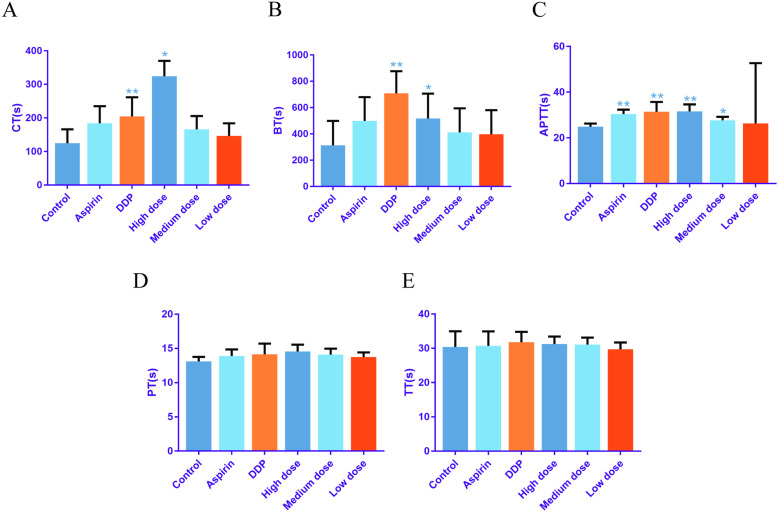
*Aspongopus chinensis* Dallas extract affects mouse coagulation time (A); Mouse tail tip bleeding time (B); Rat activated partial thromboplastin time (C); Rat thrombin time (D); Rat prothrombin time (E). Note:DDP is the Compound Danshen Dropping Pills solution group, and the high-dose, medium-dose, and low-dose groups are the *A. chinensis* extract concentration gradient group; CT is the coagulation time of ocular vein blood; BT is the blood coagulation time; APTT is activated partial thromboplastin time; PT is prothrombin time; TT is rat thrombin time; * indicates comparison with control group; * Correlation is significant at the 0.05 level; ** Correlation is significant at the 0.01 level.

*A. chinensis* extract demonstrated a notable prolonging effect on the coagulation time (CT) value and tail tip bleeding time (BT) value in mice, exhibiting a dose-response relationship with varying concentrations. The high, medium, and low dose groups exhibited CT prolongation rates of 161.3%, 33.9%, and 17.7% respectively, while the BT prolongation rates were 64.6%, 30.9%, and 26.8% respectively. Notably, the CT and BT values in the high-dose group surpassed those of the positive drug aspirin group, demonstrating the potency of *A. chinensis* extract (Fig 9A and 9B).

*A. chinensis* extract significantly extended the APTT value of rat plasma, as evidenced in Fig 9C, 9D, and 9E, indicating its prominent anticoagulant effect targeting the intrinsic coagulation pathway. While no significant impact was observed on the PT and TT values, highlighting the specificity of its anticoagulant activity. Platelet adhesion, aggregation, and release play crucial roles in the normal hemostasis process [26]. Significantly different results were observed in the medium-dose group, while the high-dose group and the positive drug group exhibited similar efficacy, with highly significant variations.

Table 5 demonstrates that *A. chinensis* extract exhibits a noteworthy inhibitory impact on ADP-induced platelet aggregation in rabbits, showcasing a dose-dependent relationship with drug concentration. At a concentration of 1.25 mg·mL^-1^, the inhibition rate parallelled that of aspirin in the positive drug group. In contrast, at a concentration of 2.5 mg·mL^-1^, the inhibition rate reached 69.22%, significantly outperforming aspirin.

**Table 5:** Effect of *Aspongopus chinensis* Dallas extract on the platelet aggregation function(‾x ± s*, n* = 6).

Group	Dose(g·mL^-1^)	Average platelet maximum aggregation rate	Inhibition rate(%)
Control	--	43.53 ± 4.25	--
Aspirin	10.00	20.14 ± 1.53 **	53.74
A. chinensis extract	0.31	42.56 ± 2.19	2.23
	0.63	39.64 ± 1.93	8.48
	1.25	19.86 ± 2.69 **	54.37
	2.50	13.4 ± 2.82 **	69.22

* indicates comparison with control group; *Correlation is significant at the 0.05 level; **Correlation is significant at the 0.01 level.

These findings indicate that the *A. chinensis* extract has anticoagulant properties *in vivo*. It has been demonstrated through coagulation function tests in rats that *A. chinensis* exerts its anticoagulant effects via the APTT pathway, targeting coagulation factors within the intrinsic coagulation pathway. Furthermore, in vitro*,* platelet aggregation experiments in rabbits have shown that the extract can influence the normal physiological functions of platelets and significantly inhibit platelet aggregation. Consequently, *A. chinensis* exhibits potent anticoagulant activity, which is likely associated with the intrinsic coagulation pathway and the inhibition of platelet aggregation.

## Discussion

After examining the chromatograms of the test samples at different elution times, we observed that the chromatogram remained stable after 30 min with no additional peaks appearing. Therefore, we selected an elution time of 30 min. We then compared four binary gradient elution systems: water (0.1% formic acid) - acetonitrile, water (0.1% formic acid) - methanol, water (0.05% trifluoroacetic acid) - acetonitrile, and water (0.05% trifluoroacetic acid) - methanol. The analysis showed that the chromatographic peak separation was optimal with water (0.05% trifluoroacetic acid) - methanol as the elution system, which also provided superior peak shapes. This system was thus determined to be the most suitable mobile phase for our study and was selected for further analyses [27]. Based on the peak shapes observed in the LC chromatograms, we continuously optimized the liquid phase conditions. Consequently, the final chromatographic and mass spectrometry conditions for the test samples were established.

Our approach to sample quality control includes measuring the content of 1,4-dihydro-4-oxoquinoline-2-carboxylic acid, as well as conducting similarity evaluation and principal component analysis through UPLC fingerprinting. Since compound **3** has the largest peak area in the HPLC fingerprint and is a common component of *A. chinensis* and its similar products, this compound was chosen as the standard reference for the fingerprint.

Four compounds were identified from *A. chinensis*, comprising nucleosides like compound **1**, xanthine derivatives such as compound **4**, and quinoline compounds, which include compound **2** and compound **3**. It is noteworthy that compound **2** and compound **4** were discovered in *A. chinensis* for the first time. Compound **1**, a nucleobase with a single-ring structure present in nucleotides [28], is a heterocyclic compound characterized by a diazine structure resulting from the replacement of two carbon atoms in a benzene ring with nitrogen atoms [29]. This modification preserves uracil’s aromaticity, similar to pyridine. Xanthine or pteridine derivatives, play an important role in a variety of metabolic pathways and extracellular processes. Enzyme pathways related to purine and pyrimidine metabolism in the human body are key biopharmaceutical targets and can be used for anti-cancer, anti-viral, anti-bacterial, and anti-inflammatory [30]. Quinoline compounds represent a significant class of nitrogen-containing heterocycles, often serving as scaffolds for various natural products, bioactive compounds, and functional materials [31]. BMS-962212, a reversible and highly selective FXIa inhibitor, has good FXIa binding affinity, anticoagulant activity, and selectivity [32]. Studies have shown that pyrroloquinoline and pyrroloisoquinoline derivatives act on a wide range of biological targets and are used as bacteriostatic, antiviral, malarial, anticancer, antidiabetic, and anticoagulant agents [33]. Quinolines are widely used in the prevention and treatment of diseases such as cancer, tumors, schizophrenia, ulcers, malaria, etc.. Drugs such as quinine and chloroquine were used by humans in the early days of the world for the prevention and treatment of malaria, and have been used ever since. In summary, it can be seen that pyrimidine and quinoline compounds have a wide range of pharmacological activities.

According to the results of anticoagulant studies, it has been concluded that *A. chinensis* contains uracil and quinoline compounds that exhibit anticoagulant effects. Yan Haiyan’s research demonstrated that guanine, compound **1**, xanthine, hypoxanthine, and adenosine, which are common medicinal ingredients found in Trichosanthis Semen and its shell and kernel, can interact with key target proteins in the *VEGF* signaling pathway (such as *NOS3*, *KDR*, and *PTGS2*) to produce anticoagulant effects [34]. This finding supports the idea that compound **1** in *A. chinensis* contributes to its anticoagulant properties and suggests that these key target proteins may mediate the anticoagulant effects of *A. chinensis* on the human body. Additionally, reports have shown that the concentrated extract of *A. chinensis* exhibits a robust fibrinolytic effect *in vitro*, which is notably higher than that of *Salvia miltiorrhiza* [35]. However, when evaluated using the fibrin plate method, none of the four compounds exhibited fibrinolytic activity. This further supports the notion that these compounds are unlikely to be responsible for the fibrinolytic effects attributed to *A. chinensis*. The fibrinolytic activity may be associated with other chemical components from *A. chinensis*, or it may result from synergistic or competitive interactions between these components. Therefore, more experiments are needed to substantiate this conclusion.

The network pharmacology results showed that compound **4** had the highest connectivity with target proteins, suggesting that compound **4** mainly interacts with core target proteins, such as *MMP9* and *PTGS2*, to produce anticoagulant effects. The full name of *MMP9* is Matrix metalloproteinase-9, which plays an important role in local proteolysis of the extracellular matrix and migration of leukocytes [36]. *MMP9* will activate substrates such as vascular endothelial growth factor (*VEGF*) and transforming growth factor *β* after cleavage, promoting angiogenesis and immune suppression [37]. *PTGS2* is an inducible enzyme that typically produces prostaglandin-like substances, which mediate responses to physiological stresses such as infection and inflammation [38]. It also catalyzes the synthesis of prostaglandins, which are integral to the regulation of blood vessel dilation and constriction, as well as influencing platelet aggregation, thereby playing a role in blood coagulation and thrombosis formation [39]. Consequently, it is hypothesized that *A. chinensis* primarily acts on *MMP9* and *PTGS2* to exert its anticoagulant effects. Additionally, the mRNA expression levels of *MMP9* and *PTGS2* were significantly reduced in LPS-induced RAW264.7 cells, further supporting this conclusion.

The APTT value measures coagulation factors in the intrinsic pathway, while the PT value measures factors in the extrinsic pathway [40,41]. The TT value reflects responses to both pathways [42]. Rat experiments show that *A. chinensis* extract affects intrinsic pathway factors via APTT. Rabbit studies reveal that this extract significantly inhibits platelet aggregation, suggesting its anticoagulant effect is linked to platelet function inhibition. Animal studies confirm *A. chinensis*’ strong anticoagulant effects, likely tied to the intrinsic pathway and platelet aggregation inhibition. *In vivo* anticoagulant experiments typically use aspirin as a positive control. Aspirin mainly inhibits platelet aggregation, unlike warfarin, which directly affects coagulation factors [43,44]. This mechanistic difference may bias conclusions when comparing the extract to aspirin. Additionally, due to dosage limitations, the compounds were not tested in animal experiments. Therefore, more experiments are needed to confirm the anticoagulant mechanism and effects of *A. chinensis*.

## Conclusions

In this study, 10 compounds were identified through UPLC-Q-TOF-MS analysis, and 4 compounds were isolated and identified from the n-butanol extract of *A. chinensis*. It is noteworthy that 6-hydroxykynurenic acid and delicatuline B were discovered in *A. chinensis* for the first time. Furthermore, PCA and PLS-DA of the UPLC fingerprint data revealed significant differences between *A. chinensis* and its similar insects. Notably, 1,4-dihydro-4-oxoquinoline-2-carboxylic acid (compound **3**) has been identified as a potential standard reference substance for content determination. The isolated compounds showed anticoagulant properties, and the mRNA expression levels of *MMP9* and *PTGS2* were significantly reduced in LPS-induced RAW264.7 cells, further supporting the network pharmacology analysis. Animal experiments confirmed the potent anticoagulant effects of *A. chinensis*, likely associated with the intrinsic coagulation pathway and the inhibition of platelet aggregation. Thus, this research provides a quality assessment method for *A. chinensis* and has also demonstrated its anticoagulant functions and substance basis.

## Supporting information

S1 File Compound structure analysis. (DOCX)

S2 Fig Nuclear Magnetic Resonance Spectroscopy (NMR) secptrum. Note:Uracil(compound 1)(A); 6-hydroxykynurenic acid(compound 2)(B); 1,4-dihydro-4-oxoquinoline-2-carboxylic acid(compound 3)(C); delicatuline B(compound 4)(D).
(TIF)

S3 Table Similarity of Aspongopus chinensis and its similar insects. Note: ACD stands for *Aspongopus chinensis* Dallas, CPD stands for *Cyclopelta parva* Distant, MI stands for *Megymenum inerme* H.-S.
(DOCX)

S4 Table The content of 1,4-dihydro-4-oxoquinoline-2-carboxylic acid in *Aspongopus chinensis* and its similar insects (‾x ± s, n = 3). Note: ACD stands for *Aspongopus chinensis* Dallas, CPD stands for *Cyclopelta parva* Distant, MI stands for *Megymenum inerme* H.-S.
(DOCX)

S5 Table Gene Ontology (GO) enrichment analysis. Note: BP represents biological process, CC represents cell composition, and MF represents biological process.
(DOCX)
