# Supplementary material for: Anticoagulant effects, substance basis, and quality assessment approach of Aspongopus chinensis Dallas
Source: PLoS One. 2025 May 14;20(5):e0320165. doi: 10.1371/journal.pone.0320165 (PMC12077788; doi:10.1371/journal.pone.0320165)
Supplement: S1 File — (DOCX) [file pone.0320165.s001.docx]

Compound structure analysis:

Uracil (Compound **1**): White solid ^1^H NMR (600 MHz, DMSO-d6) δ 7.39 (d, J = 7.6 Hz, 1H), 5.44 (d, J = 7.6 Hz, 1H).; 13C NMR (151 MHz, DMSO-d6 ) δ 164.67, 151.68, 142.53, 140.87, 100.54; the molecular weight determined by mass spectrometry is 112. After comparing the spectral data and literature, it was determined that compound 2 is uracil, with the chemical formula C_4_H_4_N_2_O_2_.

6-hydroxykynurenic acid (Compound 2): White solid ^1^H NMR (600 MHz, DMSO-d6) δ 7.83 (d, J = 8.9 Hz, 1H), 7.36 (d, J = 2.9 Hz, 1H), 7.10 (dd, J = 8.9, 2.8 Hz, 1H), 6.46 (s, 1H); 13C NMR (151 MHz, DMSO-d6) δ 177.51, 176.48, 163.53, 153.42, 133.21, 126.92, 121.81, 120.98, 106.99, 1 06.33; The molecular weight determined by mass spectrometry is 205. After comparing the spectral data and literature, it was determined that compound 3 was 6-Hydroxykynurenic acid, with the chemical formula C_10_H_7_O_4_N.

1,4-dihydro-4-oxoquinoline-2-carboxylic acid (Compound **3**): White solid ^1^H NMR (500 MHz, DMSO-d6) 8.07 (dd, J = 8.1, 1.5 Hz, 1H), 7.96 (dd, J = 8.7, 1.1 Hz, 1H), 7.68 (ddd, J = 8.5 , 6.9, 1.5 Hz, 1H), 7.34 (ddd, J = 8.0, 6.9, 1.1 Hz, 1H), 6.63 (s, 1H); 13C NMR (126 MHz, DMSO-d6) δ 177.80, 163.84, 140.64, 140.05 , 132.33, 125.74, 124.71, 123.78, 119.69, 109.53; the molecular weight determined by mass spectrometry is 189. After comparing the spectral data and literature, it was determined that compound 4 is transtorine, with the chemical formula C_10_H_7_O_3_N.

Delicatuline B (Compound **4**): White solid ^1^H NMR (500 MHz, DMSO-d6) δ 8.22 (s, 1H), 8.14 (s, 1H), 7.22 (s, 2H), 4.82 (m, J = 10.2, 8.9, 5.2 Hz, 1H), 3.12 (m, J = 16.3, 9.0 Hz, 1H), 2.94 (m, J = 16.3, 5.6 Hz, 1H), 2.11 – 1.98 (m, 1H), 1.80 (m, J = 11.0, 9.7, 8.1, 4.9 Hz, 1H), 1.18 – 1.05 (m, 1H), 1.05 – 0.90 (m, 1H), 0.81 (t, J = 7.4 Hz, 3H); 13C NMR (126 MHz, DMSO-d6) δ 172.12 , 156.18, 152.36, 149.63, 140.65, 119.22, 52.38, 39.00, 35.86, 18.93, 13.52; The mass spectrum determined the molecular weight to be 250. Comparing the spectrum data with the literature, it was determined that compound 5 was delicatuline B, with the chemical formula C_11_H_16_N_5_O_2_.
