# Supplementary figures and images for: Anticoagulant effects, substance basis, and quality assessment approach of Aspongopus chinensis Dallas
Source: PLoS One. 2025 May 14;20(5):e0320165. doi: 10.1371/journal.pone.0320165 (PMC12077788; doi:10.1371/journal.pone.0320165)

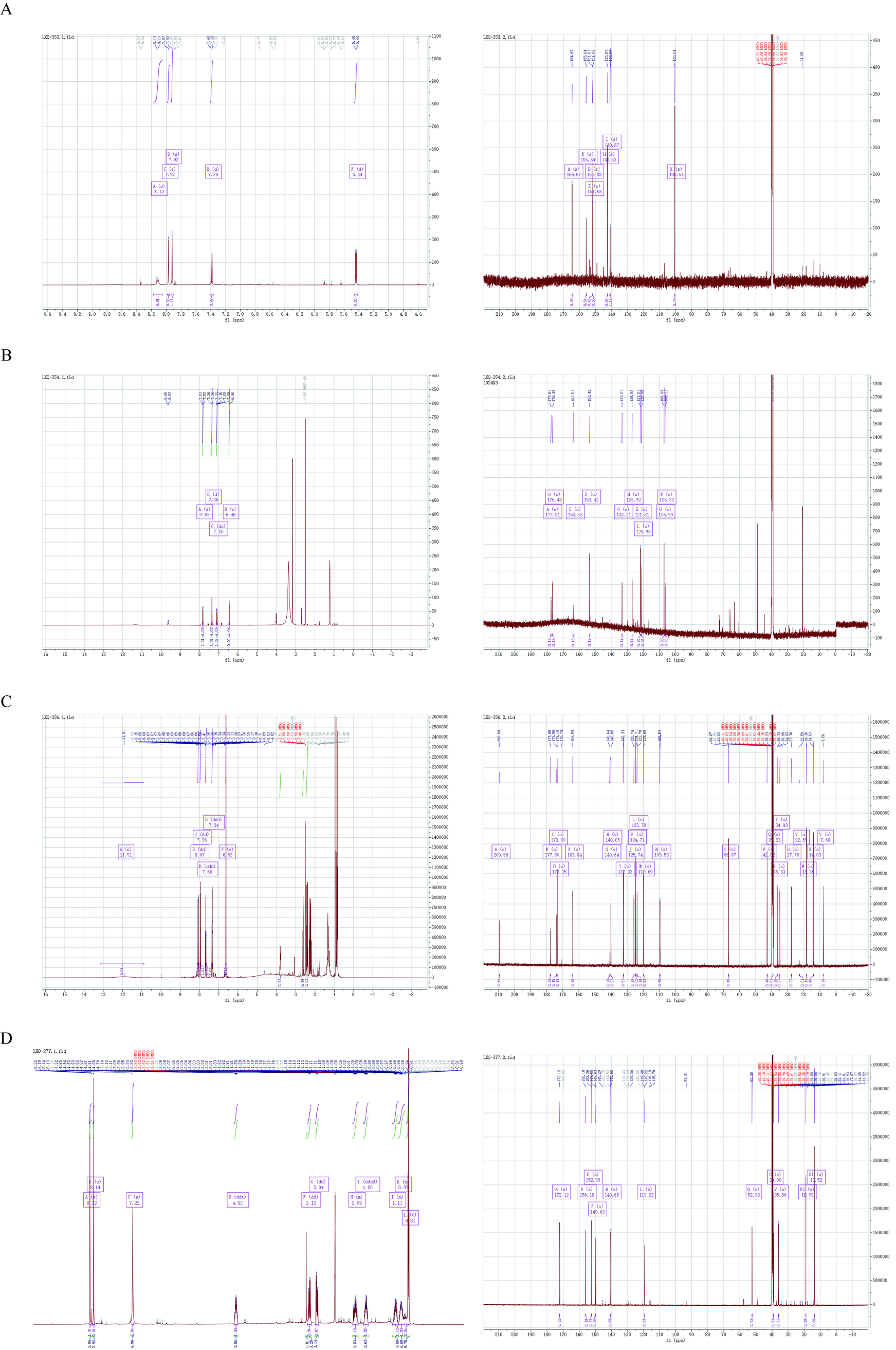

Supplement: S2 Fig — Note:Uracil(compound 1)(A); 6-hydroxykynurenic acid(compound 2)(B); 1,4-dihydro-4-oxoquinoline-2-carboxylic acid(compound 3)(C); delicatuline B(compound 4)(D). (TIF) [file pone.0320165.s002.tif]
