# Supplementary material for: Anticoagulant effects, substance basis, and quality assessment approach of Aspongopus chinensis Dallas
Source: PLoS One. 2025 May 14;20(5):e0320165. doi: 10.1371/journal.pone.0320165 (PMC12077788; doi:10.1371/journal.pone.0320165)
Supplement: S3 Table — Note: ACD stands for Aspongopus chinensis Dallas, CPD stands for Cyclopelta parva Distant, MI stands for Megymenum inerme H.-S. (DOCX) [file pone.0320165.s003.docx]

**S3 Table . Similarity of Aspongopus chinensis and its similar insects**

| **Batch number** | **Similarity** | **Batch number** | **Similarity** |
| --- | --- | --- | --- |
| ACD1- 1003 | 0.884 | ACD15-1130 | 0.933 |
| ACD2- 0506 | 0.918 | ACD16-0707 | 0.814 |
| ACD3-0502 | 0.945 | CPD1-0520 | 0.918 |
| ACD4-0509 | 0.877 | CPD2-0501 | 0.975 |
| ACD5-0319 | 0.902 | CPD3-0812 | 0.988 |
| ACD6-0559 | 0.893 | CPD4-0817 | 0.989 |
| ACD7-0508 | 0.800 | CPD5-1003 | 0.97 |
| ACD8-0507 | 0.913 | CPD6-1003 | 0.932 |
| ACD9-0510 | 0.745 | CPD7-0115 | 0.95 |
| ACD10-0512 | 0.897 | CPD8-0512 | 0.918 |
| ACD11-0508 | 0.924 | MI1-0115 | 0.829 |
| ACD12-0515 | 0.837 | MI2-0502 | 0.844 |
| ACD13-0512 | 0.884 | MI3-0424 | 0.853 |
| ACD14-0514 | 0.921 | MI4-0511 | 0.87 |

Note: ACD stands for *Aspongopus chinensis* Dallas, CPD stands for *Cyclopelta parva* Distant, MI stands for *Megymenum inerme* H.-S
