# Supplementary material for: Anticoagulant effects, substance basis, and quality assessment approach of Aspongopus chinensis Dallas
Source: PLoS One. 2025 May 14;20(5):e0320165. doi: 10.1371/journal.pone.0320165 (PMC12077788; doi:10.1371/journal.pone.0320165)
Supplement: S4 Table — Note: ACD stands for Aspongopus chinensis Dallas, CPD stands for Cyclopelta parva Distant, MI stands for Megymenum inerme H.-S. (DOCX) [file pone.0320165.s004.docx]

**S4 Table .The content of 1,4-dihydro-4-oxoquinoline-2-carboxylic acid in *Aspongopus chinensis* and its similar insects（‾x±s，n=3）**

| **Batch number** | **Content**  **(mg·g^-1^)** | **Batch number** | **Content**  **(mg·g^-1^)** | **Batch number** | **Content**  **(mg·g^-1^)** | **Batch number** | **Content (mg·g^-1^)** |
| --- | --- | --- | --- | --- | --- | --- | --- |
| ACD1- 1003 | 0.537 4±0.016 3 | ACD8-0507 | 0.530 4±0.010 8 | ACD15-1130 | 0.993 6±0.016 0 | CPD6-1003 | 0.641 1±0.015 8 |
| ACD2- 0506 | 0.650 1±0.018 2 | ACD9-0510 | 0.552 8±0.012 4 | ACD16-0707 | 0.867 0±0.009 7 | CPD7-0115 | 0.552 7±0.087 6 |
| ACD3-0502 | 0.652 1±0.020 3 | ACD10-0512 | 0.657 6±0.017 1 | CPD1-0520 | 0.434 6±0.009 7 | CPD8-0512 | 0.374 0±0.008 6 |
| ACD4-0509 | 0.553 2±0.016 7 | ACD11-0508 | 0.516 5±0.009 1 | CPD2-0501 | 0.561 2±0.019 8 | MI1-0115 | 0.607 5±0.093 5 |
| ACD5-0319 | 0.666 1±0.008 5 | ACD12-0515 | 0.508 8±0.017 2 | CPD3-0812 | 0.323 6±0.011 3 | MI2-0502 | 0.484 1±0.012 2 |
| ACD6-0559 | 0.634 4±0.010 1 | ACD13-0512 | 0.610 2±0.007 6 | CPD4-0817 | 0.364 8±0.011 6 | MI3-0424 | 0.537 9±0.016 1 |
| ACD7-0508 | 0.481 4±0.010 0 | ACD14-0514 | 0.431 5±0.008 9 | CPD5-1003 | 0.502 0±0.012 1 | MI4-0511 | 0.523 2±0.015 9 |

Note: ACD stands for *Aspongopus chinensis* Dallas, CPD stands for *Cyclopelta parva* Distant, MI stands for *Megymenum inerme* H.-S
