# Supplementary material for: Anticoagulant effects, substance basis, and quality assessment approach of Aspongopus chinensis Dallas
Source: PLoS One. 2025 May 14;20(5):e0320165. doi: 10.1371/journal.pone.0320165 (PMC12077788; doi:10.1371/journal.pone.0320165)
Supplement: S5 Table — Note: BP represents biological process, CC represents cell composition, and MF represents biological process. (DOCX) [file pone.0320165.s005.docx]

**S5 Table . Gene Ontology (GO) enrichment analysis**

| **Term** | **Gene function** | **Number of genes** | ***P value*** | **FDR** |
| --- | --- | --- | --- | --- |
| MF | endopeptidase activity | 8 | 0.00000000008 | 0.000000012 |
| BP | collagen catabolic process | 6 | 0.00000000380 | 0.000002500 |
| BP | response to beta-amyloid | 5 | 0.00000000760 | 0.000002500 |
| BP | extracellular matrix disassembly | 6 | 0.00000001000 | 0.000002500 |
| BP | response to hypoxia | 8 | 0.00000001300 | 0.000002500 |
| MF | zinc ion binding | 12 | 0.00000006800 | 0.000005100 |
| BP | proteolysis | 9 | 0.00000036000 | 0.000052000 |
| BP | cell adhesion mediated by integrin | 5 | 0.00000040000 | 0.000052000 |
| BP | aging | 7 | 0.00000053000 | 0.000059000 |
| BP | cellular response to UV-A | 4 | 0.00000063000 | 0.000062000 |
| BP | negative regulation of macrophage derived foam cell differentiation | 4 | 0.00000110000 | 0.000095000 |
| MF | metalloendopeptidase activity | 6 | 0.00000130000 | 0.000068000 |
| BP | inflammatory response | 8 | 0.00000290000 | 0.000230000 |
| BP | positive regulation of smooth muscle cell proliferation | 5 | 0.00000450000 | 0.000320000 |
| BP | extracellular matrix organization | 6 | 0.00000510000 | 0.000340000 |
| BP | regulation of blood pressure | 5 | 0.00000600000 | 0.000360000 |
| CC | integrin complex | 4 | 0.00001100000 | 0.001300000 |
| BP | endodermal cell differentiation | 4 | 0.00001200000 | 0.000690000 |
| MF | serine-type endopeptidase activity | 6 | 0.00001400000 | 0.000540000 |
| MF | G-protein coupled adenosine receptor activity | 3 | 0.00002600000 | 0.000800000 |
| BP | response to xenobiotic stimulus | 6 | 0.00003600000 | 0.001900000 |
| CC | cell surface | 8 | 0.00004000000 | 0.002400000 |
| BP | positive regulation of neuron death | 4 | 0.00004200000 | 0.002100000 |
| BP | cell migration | 6 | 0.00006000000 | 0.002800000 |
| CC | extracellular space | 12 | 0.00007400000 | 0.003000000 |
| BP | adenosine receptor signaling pathway | 3 | 0.00009100000 | 0.004000000 |
| BP | response to drug | 6 | 0.00009900000 | 0.004100000 |
| BP | negative regulation of lipid storage | 3 | 0.00011000000 | 0.004500000 |
| BP | excitatory postsynaptic potential | 4 | 0.00014000000 | 0.005100000 |
| MF | integrin binding | 5 | 0.00014000000 | 0.003500000 |
| MF | collagen binding | 4 | 0.00018000000 | 0.003700000 |
| MF | metallopeptidase activity | 4 | 0.00020000000 | 0.003700000 |
| BP | cellular response to fluid shear stress | 3 | 0.00026000000 | 0.009400000 |
| MF | virus receptor activity | 4 | 0.00026000000 | 0.004400000 |
| CC | extracellular exosome | 12 | 0.00029000000 | 0.007600000 |
| CC | plasma membrane | 18 | 0.00031000000 | 0.007600000 |
| BP | cellular protein metabolic process | 3 | 0.00034000000 | 0.011000000 |
| BP | positive regulation of smooth muscle contraction | 3 | 0.00034000000 | 0.011000000 |
| BP | prostaglandin biosynthetic process | 3 | 0.00043000000 | 0.013000000 |
| BP | cell-substrate adhesion | 3 | 0.00047000000 | 0.014000000 |
| BP | signal transduction | 9 | 0.00053000000 | 0.015000000 |
| BP | viral entry into host cell | 4 | 0.00054000000 | 0.015000000 |
| CC | external side of plasma membrane | 6 | 0.00055000000 | 0.011000000 |
| BP | positive regulation of inflammatory response | 4 | 0.00061000000 | 0.017000000 |
| CC | extracellular matrix | 5 | 0.00061000000 | 0.011000000 |
| BP | cell-matrix adhesion | 4 | 0.00066000000 | 0.017000000 |
| BP | integrin-mediated signaling pathway | 4 | 0.00070000000 | 0.018000000 |
| BP | cellular response to insulin stimulus | 4 | 0.00072000000 | 0.018000000 |
| MF | protease binding | 4 | 0.00074000000 | 0.011000000 |
| CC | extracellular region | 11 | 0.00088000000 | 0.013000000 |
| BP | heterotypic cell-cell adhesion | 3 | 0.00100000000 | 0.023000000 |
| BP | extrinsic apoptotic signaling pathway in absence of ligand | 3 | 0.00100000000 | 0.023000000 |
| BP | negative regulation of inflammatory response | 4 | 0.00110000000 | 0.026000000 |
| MF | fibronectin binding | 3 | 0.00130000000 | 0.017000000 |
| BP | positive regulation of DNA binding | 3 | 0.00130000000 | 0.028000000 |
| BP | negative regulation of apoptotic process | 6 | 0.00130000000 | 0.028000000 |
| BP | positive regulation of vasoconstriction | 3 | 0.00170000000 | 0.036000000 |
| BP | cell adhesion | 6 | 0.00180000000 | 0.036000000 |
| BP | positive regulation of apoptotic process | 5 | 0.00190000000 | 0.036000000 |
| BP | response to amphetamine | 3 | 0.00190000000 | 0.036000000 |
| BP | lung alveolus development | 3 | 0.00190000000 | 0.036000000 |
| BP | embryo implantation | 3 | 0.00210000000 | 0.038000000 |
| BP | apoptotic process | 6 | 0.00220000000 | 0.040000000 |
| BP | cellular response to beta-amyloid | 3 | 0.00260000000 | 0.046000000 |
| BP | peptidyl-serine phosphorylation | 4 | 0.00270000000 | 0.046000000 |
| BP | positive regulation of nitric oxide biosynthetic process | 3 | 0.00270000000 | 0.046000000 |

Note: BP represents biological process, CC represents cell composition, and MF represents biological process
